# Supplementary material for: Analysis of PPARγ Signaling Activity in Psoriasis
Source: Int J Mol Sci. 2021 Aug 10;22(16):8603. doi: 10.3390/ijms22168603 (PMC8395241; doi:10.3390/ijms22168603)
Supplement: Supplementary file 1 [file ijms-22-08603-s001.zip › Supplemental materials_Analysis of PPARg signaling activity in psoriasis/Pathway models/Models images and html files/Model of PPARG related pathways in psoriasis (short version)/1012571153.html]

PTK2 --+> PI3K


# ProtModification PTK2 --+> PI3K

|  |  |
| --- | --- |
| URN | urn:agi-protmodification:in-out:urn:agi-llid:5747:out:urn:agi-aopfc:0000447:eff=positive:mch=phosphorylation |
| Connectivity | 2 |
| References | 4 |
| Effect | positive |
| Mechanism | phosphorylation |

---
